# Novel Inhibitors of Mitochondrial *sn*-Glycerol 3-phosphate Dehydrogenase

**DOI:** 10.1371/journal.pone.0089938

**Published:** 2014-02-24

**Authors:** Adam L. Orr, Deepthi Ashok, Melissa R. Sarantos, Ryan Ng, Tong Shi, Akos A. Gerencser, Robert E. Hughes, Martin D. Brand

**Affiliations:** Buck Institute for Research on Aging, Novato, California, United States of America; Laurentian University, Canada

## Abstract

Mitochondrial *sn*-glycerol 3-phosphate dehydrogenase (mGPDH) is a ubiquinone-linked enzyme in the mitochondrial inner membrane best characterized as part of the glycerol phosphate shuttle that transfers reducing equivalents from cytosolic NADH into the mitochondrial electron transport chain. Despite the widespread expression of mGPDH and the availability of mGPDH-null mice, the physiological role of this enzyme remains poorly defined in many tissues, likely because of compensatory pathways for cytosolic regeneration of NAD^+^ and mechanisms for glycerol phosphate metabolism. Here we describe a novel class of cell-permeant small-molecule inhibitors of mGPDH (iGP) discovered through small-molecule screening. Structure-activity analysis identified a core benzimidazole-phenyl-succinamide structure as being essential to inhibition of mGPDH while modifications to the benzimidazole ring system modulated both potency and off-target effects. Live-cell imaging provided evidence that iGPs penetrate cellular membranes. Two compounds (iGP-1 and iGP-5) were characterized further to determine potency and selectivity and found to be mixed inhibitors with IC_50_ and *K*_i_ values between ∼1–15 µM. These novel mGPDH inhibitors are unique tools to investigate the role of glycerol 3-phosphate metabolism in both isolated and intact systems.

## Introduction

**Figure 1:**
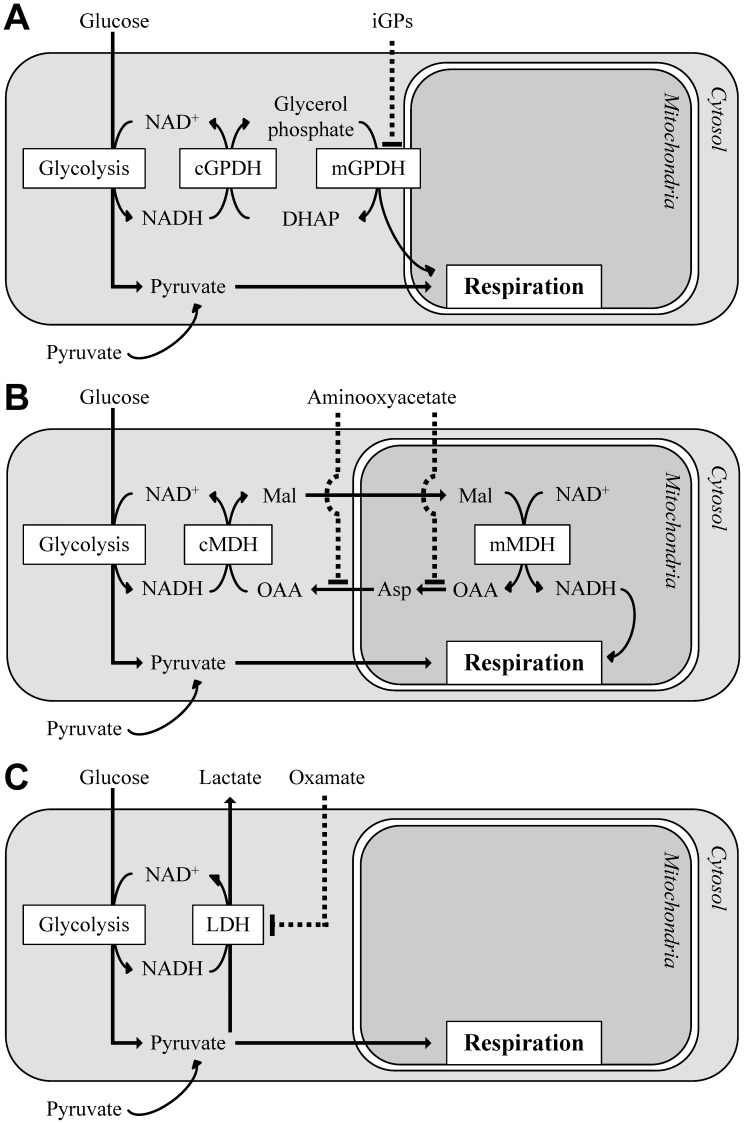
The glycerol phosphate shuttle is one of several pathways to regenerate cytosolic NAD^+^ for glycolysis. Glycolytic metabolism of glucose to pyruvate involves the reduction of cytosolic NAD^+^ to NADH at the step catalyzed by glyceraldehyde 3-phosphate dehydrogenase. Several mechanisms exist to regenerate cytosolic NAD^+^ to ensure that a high NADH/NAD^+^ ratio does not limit glucose metabolism. (**A**) The glycerol phosphate shuttle comprises soluble, NAD^+^-linked cGPDH and the membrane bound, FAD-linked mGPDH. This system regenerates cytosolic NAD^+^ and transfers the reducing equivalents directly into the mobile ubiquinone pool of the mitochondrial electron transport chain. (**B**) The malate-aspartate shuttle comprises multiple enzymes and mitochondrial carriers that interconvert and transport dicarboxylates and amino acids. In the cytosol, malate dehydrogenase regenerates NAD^+^ during reduction of oxaloacetate to malate. This malate is transported into the mitochondrial matrix where it is oxidized back to oxaloacetate by mitochondrial malate dehydrogenase, producing NADH that is reoxidized by complex I of the electron transport chain. To complete the shuttle, cytosolic oxaloacetate is regenerated by sequential transaminations through aspartate. These transaminations are potently inhibited by aminooxyacetate. *cMDH*, cytosolic malate dehydrogenase; *mMDH*, mitochondrial malate dehydrogenase; *OAA*, oxaloacetate; *Mal*, malate; *Asp*, aspartate (**C**) Lactate dehydrogenase regenerates cytosolic NAD^+^ at the expense of mitochondrial oxidation of pyruvate. *LDH*, lactate dehydrogenase. In certain systems, such as neuronal synapses, the maintenance of cytosolic NAD^+^ by each of these mechanisms can be made less important by direct uptake and oxidation of pyruvate.

Mitochondrial *sn*-glycerol 3-phosphate dehydrogenase (mGPDH^3^; EC 1.1.5.3; gene symbol *GPD2*) is an important link between cytosolic and mitochondrial energy transduction. mGPDH is a ubiquinone-linked flavoprotein embedded in the outer leaflet of the mitochondrial inner membrane that transfers reducing equivalents directly from glycerol 3-phosphate into the electron transport chain [1], [2]. Glycerol 3-phosphate is an intermediate common to both lipid and carbohydrate metabolism. Its oxidation to dihydroxyacetone phosphate (DHAP) by mGPDH and the subsequent reduction of DHAP back to glycerol 3-phosphate by a distinct soluble GPDH (cytosolic GPDH; cGPDH; EC 1.1.1.8; gene symbol *GPD1*) regenerates NAD^+^ consumed during glycolysis (Fig. 1A). This cyclic process of transferring reducing equivalents from cytosolic NADH into the mitochondrial respiratory chain is known as the glycerol phosphate shuttle. Along with the malate-aspartate shuttle (Fig. 1B) and production of lactate by lactate dehydrogenase (Fig. 1C), it is a common mechanism by which cytosolic NAD^+^ is regenerated to facilitate glycolytic activity in a variety of cell types [3].

The active site of mGPDH faces the mitochondrial intermembrane space, as does its calcium-sensitive EF-hand domain that lowers the *K*_m_ for glycerol 3-phosphate as physiological levels of free calcium rise [1], [2], [4], [5]. This orientation is thought to allow mGPDH to coordinate cytosolic and mitochondrial metabolism during periods of high activity and, not surprisingly, mGPDH is expressed most highly in tissues with variable energy demands including thermogenic brown fat, type II skeletal muscle fibers, brain, sperm and pancreatic *β*-cells [6], [7]. Further, mGPDH expression is hormonally regulated to alter tissue activity both during development and in response to environmental challenges [8]–[10]. Despite the widespread expression of the enzyme, mGPDH-knockout mice display relatively mild phenotypes beyond weaning. These include decreased body mass and decreased white fat mass. However food intake, non-white fat tissues, and metabolic profiles are normal in these mice [11]. Prior to weaning, viability of mGPDH-null pups is decreased by 50%. Such a dramatic developmental bottleneck raises the possibility that the absence of mGPDH in surviving adults may be successfully compensated for by parallel metabolic pathways. In fact, further roles for mGPDH have only been observed after additional genetic, pharmacologic, or environmental manipulations. For example, ablation of cGPDH as well as mGPDH prevents compensatory responses in glycerol 3-phosphate metabolism, causes dramatic changes in metabolic profiles, and is lethal within one week of birth [12].

An alternative strategy to genetic manipulation of enzyme expression is the acute use of selective inhibitors. Several groups have demonstrated inhibition of mGPDH or bacterial GPDH by various small metabolite analogs of glycerol 3-phosphate [2], [13]–[15]. These analogs, the most potent being the glycolytic intermediate glyceraldehyde 3-phosphate, act by competitive inhibition in the substrate-binding pocket [2]. DHAP is both a glycolytic intermediate and product of mGPDH. Whereas NAD^+^-linked cGPDH can interconvert DHAP and glycerol 3-phosphate, the ubiquinone-linked reaction catalyzed by mGPDH is irreversible and displays product inhibition by DHAP [2]. Additionally, lipid metabolites such as free fatty acids and acyl CoAs, inorganic ions such as sulfate and phosphate, and several heavy metal ions also inhibit FAD-linked GPDH activity [2], [15]–[17]. Importantly, many of these are not cell-permeable and none is selective for mGPDH. Other proposed small-molecule inhibitors of mGPDH activity such as polyborates, indomethacin, doxorubicin, or diazoxide are also not selective [6], [18]–[23]. Therefore, a need remains for selective and cell-permeant inhibitors of mGPDH to elucidate the role of this widely-expressed enzyme under appropriate physiological conditions [3], [24]. Here we describe the identification and characterization of a novel class of small-molecule inhibitors of mGPDH activity (iGP) that are cell-permeant, potent, and highly selective for mGPDH.

## Materials and Methods

### Reagents

CaCl_2_ standard was from Thermo Scientific, fatty acid-free BSA from Calbiochem, and atpenin A5 from Santa Cruz Biotechnology. Amplex UltraRed, tetramethylrhodamine methyl ester (TMRM), LysoTracker Red DND-99, and Geltrex were from Invitrogen. Compounds for screening were arbitrarily selected from a diverse, non-combinatorial library obtained from ChemBridge (∼10 mM in DMSO). New stocks of primary hits and their structural analogs were also sourced from ChemBridge (and dissolved to 80 mM in DMSO). The biological activity of iGP-1 (ChemBridge ID 5224148) was validated using compound obtained from Vitas-M (ID STK017597) and found to be essentially identical. All other reagents were from Sigma-Aldrich. *sn*-Glycerol 3-phosphate was added as disodium *rac*-α/β-glycerol phosphate (25% active optical isomer *sn*-glycerol 3-phosphate, 25% inactive optical isomer *sn*-glycerol 1-phosphate and 50% inactive structural isomer glycerol 2-phosphate) and will be referred to as glycerol phosphate. Where indicated, total and free calcium concentrations in assay buffers were calculated using the Extended MaxChelator program available through Chris Patton on the Stanford University Web site at http://maxchelator.stanford.edu.

### Animals

Skeletal muscle mitochondria were isolated from hindlimbs of 5–8 week old female Wistar rats (Harlan Laboratories) as previously described [25]. Cortical synaptosomes were isolated from 6–12 week old male C57BL/6 mice (Jackson Labs) as previously described [26]. The animal protocol was approved by the Buck Institute Animal Care and Use Committee, in accordance with IACUC standards.

### Measurement of Mitochondrial H_2_O_2_ Production and Membrane Potential

Superoxide was measured as H_2_O_2_ after dismutation by endogenous or exogenous superoxide dismutase. Rates of total superoxide/H_2_O_2_ production were measured fluorimetrically using Amplex UltraRed in the presence of exogenous superoxide dismutase and horseradish peroxidase in a Varian Cary Eclipse fluorimeter as previously described [27]. Rates of production from specific sites were determined as previously described using the following eight conditions: flavin mononucleotide site of complex I (site I_F_) plus matrix NAD-linked dehydrogenases (collectively "site I_F_/DH") with 5 mM glutamate, 5 mM malate, and 4 µM rotenone [28]; ubiquinone-binding site of complex I (site I_Q_) with either 0.5 or 5 mM succinate [29]; flavin site of complex II (site II_F_) with either 15 µM palmitoylcarnitine, 2.5 µM antimycin A, and 2 µM myxothiazol or 0.5 mM succinate, 4 µM rotenone, 2.5 µM antimycin A, and 2 µM myxothiazol [30], [31]; mGPDH with 1.7 mM glycerol phosphate, 4 µM rotenone, 2.5 µM antimycin A, 2 µM myxothiazol, 1 mM malonate, and 250 nM free calcium; outer ubiquinone-binding site of complex III (site III_Qo_) with either 0.5 or 5 mM succinate, 4 µM rotenone and 2.5 µM antimycin A [32]. Candidate small-molecule inhibitors were added prior to the start of measurements and H_2_O_2_ calibration curves were performed in the presence of tested compounds.

Mitochondrial membrane potential (ΔΨm) was estimated fluorimetrically as described previously [33] on the Varian fluorimeter, except the potentiometric dye TMRM was used in quench-mode in place of safranine O [34]. Briefly, skeletal muscle mitochondria (0.2 mg protein ⋅ mL^−1^) were incubated with 5 µM TMRM in the presence of inhibitors or vehicle. ΔΨm was generated by sequential addition of substrates of complex I (5 mM glutamate plus 5 mM malate), complex II (4 µM rotenone then 0.5 or 5 mM succinate), and mGPDH (2 µM atpenin A5 then 1.7 or 16.7 mM glycerol phosphate). The effect of putative small-molecule inhibitors was normalized as the % change in fluorescence between vehicle treatment and complete depolarization by the uncoupler carbonyl cyanide-4-(trifluoromethoxy)phenylhydrazone (FCCP, 2 µM) added at the end of each run. When indicated, the K^+^/H^+^ exchanger nigericin (80 ng ⋅ ml^−1^) was added to collapse the pH gradient across the mitochondrial inner membrane (ΔpH) and maximize ΔΨm.

The assay buffer for both H_2_O_2_ and ΔΨm measurements was 120 mM KCl, 5 mM HEPES, 1 mM EGTA, 0.3% (w/v) BSA without or with 250 nM free calcium at pH 7.0. All assays were performed with stirring at 37°C.

### Measurement of iGP-1 Fluorescence in Solution

Excitation and emission spectra of iGP-1 fluorescence were acquired with the Varian fluorimeter in standard buffer with pH adjusted to between 1.5 and 11 as indicated.

### Screening for Inhibitors of H_2_O_2_ Production by mGPDH

3,200 compounds were tested initially at 2.5 µM (0.025% DMSO final) in duplicate on different plates against a panel of five distinct assays of site-specific superoxide/H_2_O_2_ production as described in the previous section with minor changes. Targeted sites included site I_F_/DH with 5 mM glutamate, 5 mM malate, and 4 µM rotenone, site I_Q_ with 5 mM succinate, site II_F_ with 15 µM palmitoylcarnitine, 2.5 µM antimycin A, and 2 µM myxothiazol, mGPDH with 16.7 mM glycerol phosphate, 4 µM rotenone, 2.5 µM antimycin A, 2 µM myxothiazol, 1 mM malonate, and no added calcium, and site III_Qo_ with 5 mM succinate, 4 µM rotenone and 2.5 µM antimycin A. Compounds and assay components were distributed by a Biomek FX liquid handling workstation (Beckman) into 96-well black plates (Nunc) to a final volume of 200 µL, and incubated in the dark at room temperature for 30–40 min after the addition of substrate. Endpoint fluorescence was measured on a Perkin Elmer Victor V3 fluorescent plate reader. The fluorescence intensity of each well was normalized as % change from the average of eight DMSO controls on the same plate. Duplicate wells of each of four modulators of mitochondrial metabolism known to inhibit specific sites of superoxide/H_2_O_2_ production were included on each plate as positive controls and the resulting fluorescence levels set as −100% change from DMSO controls. Known inhibitors included 20 mM aspartate (for site I_F_/DH), 1 µM FCCP (for site I_Q_), 10 mM malonate (for site II_F_), and 2 µM myxothiazol (for site III_Qo_). The background fluorescence of the FCCP wells in the site I_Q_ assay was used to normalize mGPDH plates run in parallel. To minimize position-dependent effects across each assay plate, Tukey’s two-way median polish was applied to fluorescence values after initial normalization [35], [36]. Tukey’s polish is an iterative subtraction of the median row and column values from each well’s given row or column. The procedure is repeated until sequential medians for each row and column are zero and positional gradients in fluorescence are eliminated.

Novel inhibitors of superoxide/H_2_O_2_ production by mGPDH were identified as compounds whose average effect was to decrease H_2_O_2_ production by at least 10% without altering H_2_O_2_ production in other assays by more than ±10%. Select hits in the mGPDH assay were re-sourced from ChemBridge and retested at 0.08–80 µM (0.1% DMSO final) in duplicate on the same microplate as described above against the eight conditions listed under “Measurement of mitochondrial H_2_O_2_ production and membrane potential.”

### Analysis of Structure/activity Relationships

Structure/activity analysis was performed on 18 structural analogs of two structurally-related hits from the primary screen (iGP-1 and iGP-2). These 20 compounds were tested in duplicate at 0.08–80 µM (0.1% DMSO final) in microplate format for effects on site-specific H_2_O_2_ production as described above. Additionally, four microplate assays for ΔΨm were run in parallel to the H_2_O_2_ assays. These assays were as described under “Measurement of mitochondrial H_2_O_2_ production and membrane potential” except that each substrate condition was assayed in a separate microplate. Compounds were tested against the following substrate conditions: 5 mM glutamate and 5 mM malate both without and with 80 ng ⋅ mL^−1^ nigericin, 5 mM succinate and 4 µM rotenone with 80 ng ⋅ mL^−1^ nigericin, and 16.7 mM glycerol phosphate and 4 µM rotenone with 80 ng ⋅ mL^−1^ nigericin. Plates were incubated in the dark at room temperature for 30–40 min (H_2_O_2_) or 10–15 min (ΔΨm) after the addition of substrate, and endpoint fluorescence was measured on the Victor plate reader. Endpoints were chosen for the H_2_O_2_ assays to provide good signal-to-noise ratios and linear rates of change without the indicator dye limiting the reaction. Endpoints for the ΔΨm assays were determined in pilot experiments as the times for TMRM accumulation to reach a steady state. At room temperature, this was typically ∼ 5 min and remained stable for at least 30 min. Endpoint fluorescence values were normalized as described in the previous section with wells containing FCCP serving as positive controls for the ΔΨm assays.

### cGPDH and mGPDH Activity

The effects of small-molecule inhibitors on cGPDH and mGPDH enzymatic activity were tested according to established procedures. Activity of purified rabbit muscle cGPDH was measured as described by the manufacturer as the rate of oxidation of NADH in the presence of DHAP on the Varian fluorimeter (λ_ex_ = 365 nm, λ_em_ = 450 nm). Activity of cGPDH in frozen-thawed synaptosomes was measured at 37°C in a BMG Labtech PHERAstar Plus microplate reader as the linear rate of change in NADH absorbance (λ = 340 nm) specific to the addition of 2.5 mM DHAP and insensitive to 4 µM rotenone. Rates were converted to nmol NADH using an extinction coefficient of 6.22 mM^−1^ ⋅ cm^−1^ and a calculated pathlength of 0.6 cm.

Activity of mGPDH in frozen-thawed skeletal muscle mitochondria was measured as 2,6-dichlorophenolindophenol (DCPIP)-linked reduction by glycerol phosphate as described previously in the presence of 1 µM free calcium [27]. Linear rates of change in absorbance (λ = 600 nm) were measured on the PHERAstar microplate reader and converted to rates of DCPIP reduction using an extinction coefficient of 21 mM^−1^ ⋅ cm^−1^ and a calculated pathlength of 0.6 cm. Kinetic parameters and inhibitor constants were determined by co-varying glycerol phosphate and novel inhibitors according to recommended procedures [37] and calculated for each data set using GraphPad Prism. Vmax and Km were determined using hyperbolic curves fit to the Michaelis-Menten equation: rate = (Vmax ⋅ [substrate]) ⋅ (*K*_m_+[substrate])^ −1^. Hill Slope and IC_50_ were determined using unconstrained, sigmoidal dose-response curve fits of % remaining activity against concentration of iGP. *K*_ic_ and *K*_iu_ were determined using plots of rate^−1^ *vs* iGP and GP ⋅ rate^−1^ vs iGP, respectively [37]. Linear regression analysis yielded an average *x*-coordinate at which the best-fit lines intersect. These *x*-coordinates correspond to -*K*_ic_ and -*K*_iu_ in their respective plots [37]. The apparent Vmax of mGPDH is dependent upon the concentration of DCPIP used [13], [38]. Therefore, to determine the maximal activity of mGPDH in isolated synaptosomes, DCPIP was replaced by 1 mM potassium ferricyanide [39] and linear rates of change in absorbance (λ = 420 nm) were converted to rates of ferricyanide reduction using an extinction coefficient of 1.04 mM^−1^ ⋅ cm^−1^ and a calculated pathlength of 0.6 cm.

### Mitochondrial and Synaptosomal Respiration

The effects of small-molecule inhibitors on respiration were tested in plate-attached skeletal muscle mitochondria using a Seahorse XF24 Analyzer according to published protocols [40]. Briefly, mitochondria (2 or 4 µg protein) were attached to Seahorse assay plates by centrifugation in a mannitol and sucrose-based medium (Seahorse MAS buffer [40]) containing 0.3% (w/v) BSA without or with 250 nM free calcium at pH 7.0. Compounds were titrated to 80 µM on each of four parallel plates with media containing one of the following substrates: 10 mM pyruvate and 0.5 mM malate; 5 mM glutamate and 5 mM malate; 5 mM succinate and 4 µM rotenone; or 16.7 mM glycerol phosphate and 4 µM rotenone with 250 nM free calcium. Compounds were added in these media just prior to loading the assay plate into the Seahorse instrument. Additions were made to set respiration in basal state 2 (substrate only), phosphorylating state 3 (5 mM ADP), non-phosphorylating state 4o (0.5 ug ⋅ mL^−1^ oligomycin), and to reveal the non-mitochondrial rate (2 µM myxothiazol and 2.5 µM antimycin A). Each concentration of compound was tested in at least two wells on each plate. At least three biological replicates of each titration in each substrate condition were performed.

Synaptosomal respiration was tested in the Seahorse XF24 using published protocols [26], [41]. Synaptosomes (20 or 25 µg protein) were attached to polyethyleneimine and Geltrex-coated Seahorse plates by centrifugation in ionic medium [26], [41] containing 10 mM glucose. Attachment buffer was carefully replaced with ionic buffer containing DMSO, 100 µM iGP-1**,** 0.5 mM aminooxyacetate, or iGP-1 with aminooxyacetate, and either 10 mM pyruvate or 15 mM glucose. Plates were equilibrated for 20 min at 37°C then loaded into the instrument followed by measurement of basal respiration and injection of 5 µM FCCP (to induce maximal, uncoupled respiration) with 4 µg ⋅ mL^−1^ oligomycin (to prevent compensatory reversal of ATP synthase). In some experiments, 10 mM oxamate was added with the FCCP/oligomycin to minimize regeneration of cytosolic NAD^+^ by lactate dehydrogenase. Inhibition of the malate-aspartate shuttle was defined as the difference between maximal rates ± aminooxyacetate, expressed as % of control. Inhibition of the glycerol phosphate shuttle was the difference between maximal rates with aminooxyacetate ± iGP-1, as % of control.

### Live Cell Imaging

The ability of iGP-1 to cross cellular membranes was evaluated in STHdhQ7 cells cultured at 33°C as described [42]. Cells were seeded on collagen-coated, Lab-Tek chambered coverglass (Nunc) 48 h before use. Prior to imaging, cells were cultured for 45 min with 65 nM LysoTracker Red ±250 nM bafilomycin A1. Medium was then replaced with imaging buffer (120 mM NaCl, 3.5 mM KCl, 0.4 mM KH_2_PO_4_, 20 mM TES, 5 mM NaHCO_3_, 1.2 mM Na_2_SO_4_, 1.5 mM CaCl_2_, 0.5 mM MgCl_2_, 2 mM Glucose, and 0.2% BSA, pH 7.4) containing DMSO, 250 nM bafilomycin A1 or 100 µM iGP-1. Cells were imaged on a Nikon Eclipse Ti-PFS inverted microscope equipped with a Cascade 512B camera (Photometrics, Tucson, AZ), a Plan Fluor 100X/1.3 oil objective, an LB-LS17 Xe-arc light source (attenuated), 10–3 excitation and emission filter wheels (Sutter Instruments, Novato, CA), and an MS-2000 linear encoded motorized stage (ASI, Eugene, OR). The filter sets, given as excitation, dichroic mirror, emission in nm/bandwidth, were, for iGP-1 (1 s exposure), 340/26, 409, 460/80 and for LysoTracker Red (100 ms exposure), 543/22, 562, 588/21 (all from Semrock, Rochester, NY). Image acquisition was controlled by NIS Elements 4.0 (Nikon, Melville, NY). Images of cell-free wells containing buffer with DMSO or iGP-1 were used for background subtraction in Image Analyst MKII (Novato, CA).

### Data Analysis

Data are presented as mean ± S.E. or mean ±95% C.I. where indicated. Statistical differences between conditions were analyzed by one-way ANOVA with Newman-Keuls post-test as specified in the figure legends. *p* values <0.05 were considered significant.

## Results

### Selectivity of Known Inhibitors of mGPDH

**Figure 2:**
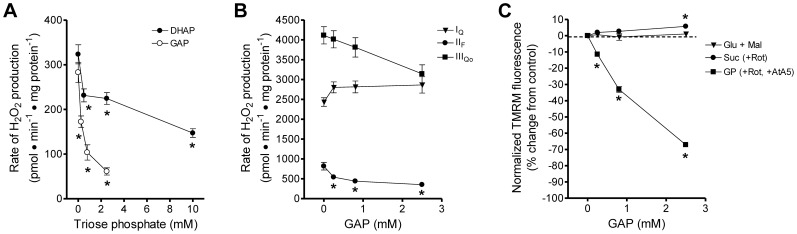
Small-metabolite inhibitors of mGPDH are not selective. (**A**) Effect of DHAP (*black circles*) and glyceraldehyde 3-phosphate (*white circles*) on the rate of H_2_O_2_ production in the presence of 1.7 mM glycerol phosphate, 4 µM rotenone, 2 µM myxothiazol, 2.5 µM antimycin A, and 250 nM free calcium. The rate of H_2_O_2_ production by mGPDH was significantly inhibited by each triose phosphate (*p<0.05 *versus* buffer control; one-way ANOVA with Newman-Keuls post-test). Data are means ± S.E. (n = 3). (**B**) Effect of glyceraldehyde 3-phosphate on the rate of H_2_O_2_ production from site I_Q_ (*triangles*; 5 mM succinate), site II_F_ (*circles*; 0.5 mM succinate, 4 µM rotenone, 2 µM myxothiazol, 2.5 µM antimycin A), and site III_Qo_ (*squares*; 5 mM succinate, 3 mM malonate, 4 µM rotenone, 2.5 µM antimycin A). The rate of H_2_O_2_ production by site II_F_ was significantly inhibited (*p<0.05 *versus* buffer control; one-way ANOVA with Newman-Keuls post-test). Data are means ± S.E. (n = 3). (**C**) Effect of glyceraldehyde 3-phosphate on ΔΨm powered by 5 mM glutamate and 5 mM malate (*triangles*), 5 mM succinate and 4 µM rotenone (*circles*), or 16.7 mM glycerol phosphate with 4 µM rotenone and 2 µM atpenin A5 (*squares*) all in the presence of 80 ng ⋅ ml^−1^ nigericin to collapse ΔpH. ΔΨm powered by glycerol phosphate was significantly decreased at all concentrations of glyceraldehyde 3-phosphate tested. ΔΨm powered by succinate was significantly increased at 2.5 mM glyceraldehyde 3-phosphate. (*p<0.05 *versus* buffer control; one-way ANOVA with Newman-Keuls post-test). Data are means ± S.E. (n = 3). *GAP*, glyceraldehyde 3-phosphate; *Glu*, glutamate; *Mal*, malate; *Suc*, succinate; *Rot*, rotenone; *GP*, glycerol phosphate; *AtA5*, atpenin A5. When not visible, error bars are obscured by the symbol.

Analogs of glycerol phosphate, including glyceraldehyde 3-phosphate and DHAP, are established competitive inhibitors of bacterial, invertebrate, and vertebrate homologs of FAD-linked GPDH [2], [13]–[15]. Because of their limited ability to cross membranes and their involvement in cellular metabolism, these inhibitors are not useful in intact cells. However, neither glyceraldehyde 3-phosphate nor DHAP is oxidized by mitochondria [14] and therefore they may be useful as selective inhibitors of mGPDH in isolated mitochondria. To test this possibility, we measured their effects on the rates of H_2_O_2_ production and levels of ΔΨm in mitochondria provided with different substrates (Fig. 2).

Consistent with their known effects on mGPDH enzymatic activity [2], [13]–[15], both glyceraldehyde 3-phosphate and DHAP inhibited the rate of H_2_O_2_ production from mGPDH (Fig. 2A). Glyceraldehyde 3-phosphate was more potent, inhibiting nearly 80% at 2.5 mM, but DHAP was less effective. The selectivity of glyceraldehyde 3-phosphate was tested by measuring its effect on H_2_O_2_ production from site I_Q_, site II_F_, and site III_Qo_. It significantly inhibited the rate of H_2_O_2_ production from site II_F_ (Fig. 2B), suggesting a lack of specificity.

As expected, glyceraldehyde 3-phosphate also lowered ΔΨm driven by glycerol phosphate oxidation (Fig. 2C). To test its selectivity in this assay, we also measured ΔΨm driven by glutamate plus malate and by succinate. ΔΨm driven by succinate was significantly increased. This increase in ΔΨm was independent of changes in ΔpH since the K^+^/H^+^ exchanger nigericin was present in all conditions.

We conclude that glyceraldehyde 3-phosphate is not a specific inhibitor of mGPDH but also alters succinate oxidation. Therefore, even in isolated mitochondria, small metabolite inhibitors of mGPDH are limited in utility.

### Discovery of Novel Small-molecule Inhibitors of Superoxide/H_2_O_2_ Production by mGPDH

**Figure 3:**
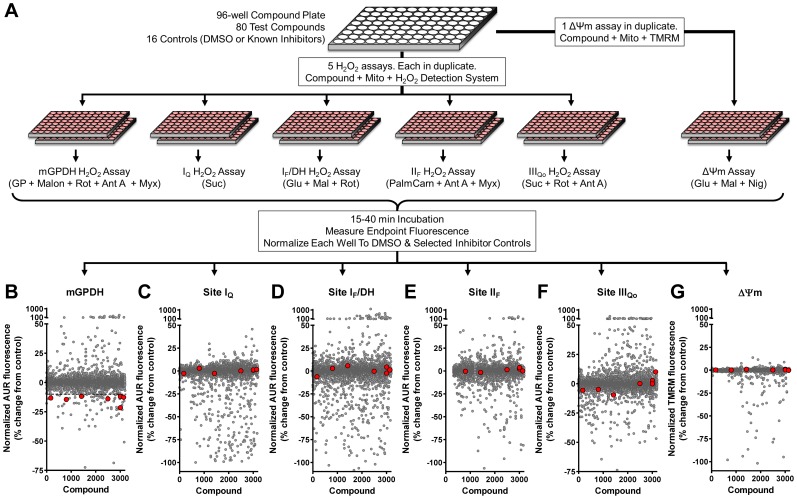
Screening design for novel inhibitors of mitochondrial H_2_O_2_ production. (**A**) Screening workflow. Compounds were screened in duplicate in microplates against six assays. Five assays each targeted a distinct site of superoxide/H_2_O_2_ production using different substrates without or with inhibitors. The sites assayed were site I_Q_, site I_F_/DH, site II_F_, site III_Qo_, and mGPDH. A sixth assay of ΔΨm was used to eliminate general inhibitors of mitochondrial function. All assays were initiated by the addition of Start Solutions containing the substrates and inhibitors listed in parentheses. Details are in “Materials and Methods” and reference 43. Endpoint fluorescence was measured and the effect of compounds was scaled to positive and negative controls included on each assay plate. (**B – G**) Average normalized effects of 3200 compounds screened against all six assays in duplicate (gray circles). 87 compounds gave >10% inhibition of mGPDH superoxide/H_2_O_2_ production (dashed line in B). However, after evaluating their effects in the other screens, only 7 were selective for superoxide/H_2_O_2_ production from mGPDH and did not impair ΔΨm driven with glutamate and malate (red circles in B – G). AUR, resorufin product of Amplex UltraRed oxidation; Suc, succinate; Glu, glutamate; Mal, malate; PalmCarn, palmitoylcarnitine; GP, glycerol 3-phosphate; Rot, rotenone; Ant A, antimycin A; Myx, myxothiazol; Malon, malonate; Nig, nigericin.

We recently described a multiple-parallel screen for novel modulators of mitochondrial H_2_O_2_ production [43]. Each assay in the screen was designed to either drive superoxide/H_2_O_2_ production predominantly from one mitochondrial redox center or to report undesired effects on ΔΨm (Fig. 3A). Included in this screen was an assay specific for superoxide/H_2_O_2_ production by mGPDH. This assay was robust with a %CV for intraplate DMSO control wells of 3.4±0.2% (n = 40 plates each tested in duplicate). Out of 3200 compounds arbitrarily selected from a structurally diverse chemical library and tested at 2.5 µM, 87 (2.7%) decreased the signal in the mGPDH assay by at least 10% compared to DMSO controls (Table S1). To identify compounds that were selective for mGPDH, these 87 compounds were counter-screened using a panel of four assays that each targeted a distinct site of superoxide/H_2_O_2_ production: site I_F_/DH, site I_Q_, site II_F_, and site III_Qo_. Compounds that increased or decreased the signal more than 10% in any of these assays were eliminated, leaving seven (0.2%) selective mGPDH inhibitors (Fig. 3B–3G).

**Figure 4:**
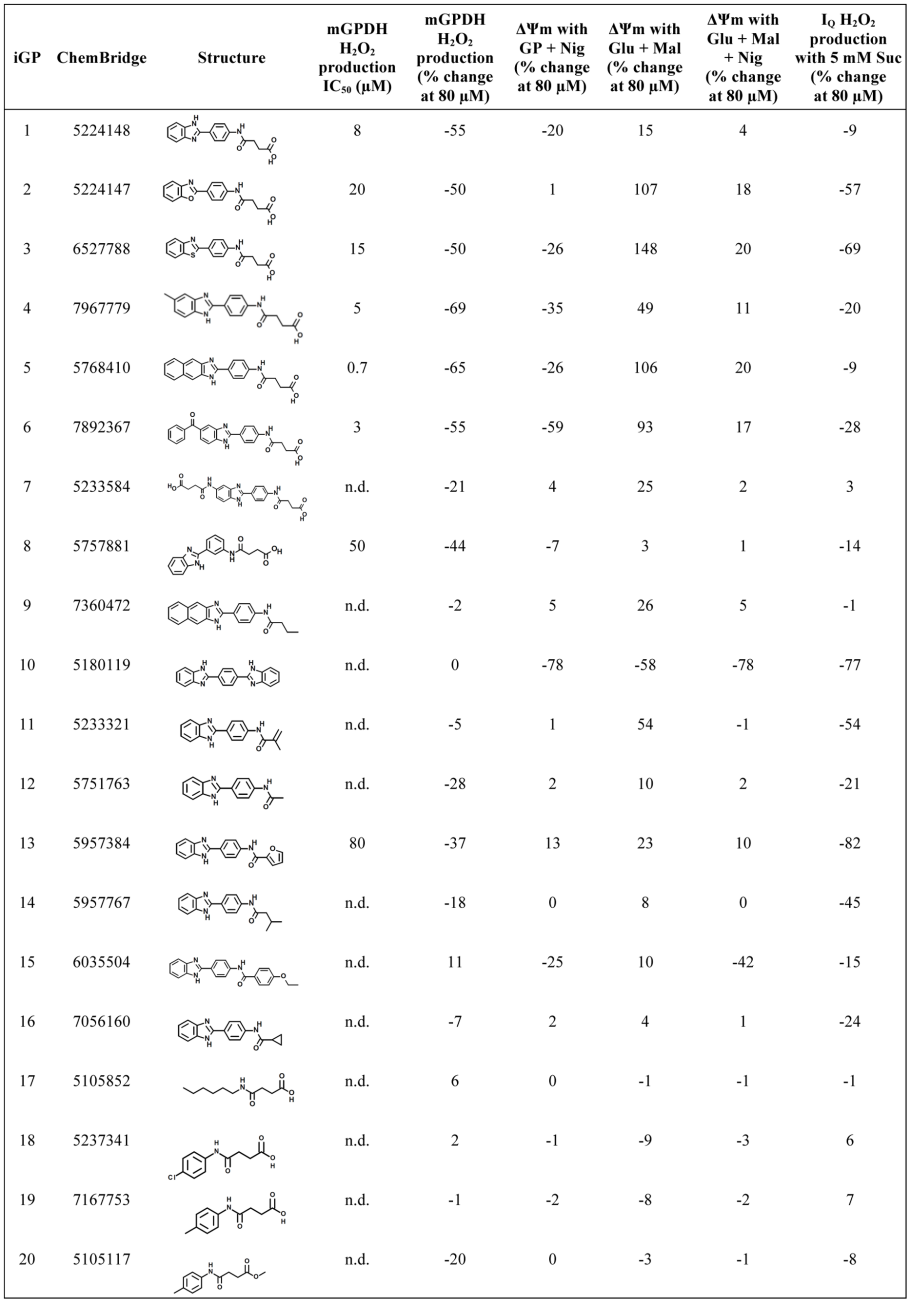
Structure/activity analysis identifies features conferring mGPDH inhibition. Summary of effects on H_2_O_2_ production and ΔΨm of 20 compounds structurally related to the top hit in our primary screen. Each compound was tested against eight assays of site-selective H_2_O_2_ production and four assays of ΔΨm powered by different mitochondrial substrates (see Materials and Methods for details of individual assays). Data are means of two replicates on the same plate. Five criteria best segregated these compounds according to changes in structural motifs relative to the parent compound iGP-1: potency of inhibition of mGPDH H_2_O_2_ production (defined by estimating the IC_50_ concentration or, where an IC_50_ could not be calculated, the effect on mGPDH H_2_O_2_ production at 80 µM), effect on ΔΨm powered by glycerol phosphate, effect on ΔΨm powered by glutamate plus malate in the absence or presence of the K^+^/H^+^ exchanger nigericin, and effect on H_2_O_2_ production by site I_Q_ driven by succinate. Note that because the method used to normalize mGPDH H_2_O_2_ production in the screening assay underestimated the background rate, the maximal % inhibition for this assay was ∼70% (see Materials and Methods). *Glu*, glutamate; *Mal*, malate; *Nig*, nigericin; *Rot*, rotenone; *GP*, glycerol phosphate; *Suc*, succinate.

We subsequently obtained four of the most potent and selective of these compounds from the original supplier and retested each over a broad concentration range (0.08–80 µM) against a panel of eight assays of H_2_O_2_ production targeting the five sites listed above using additional substrate conditions to better evaluate selectivity and potential mechanisms of action. Two compounds were false positives. The other two (iGP-1 and iGP-2; ChemBridge #5224148 and #5224147; Fig. 4) differed structurally by a single atom and each retested well. Another of the seven hits, #5244345, was also structurally similar to these two but displayed less selectivity and was not retested. The most potent compound in the initial screen against H_2_O_2_ production by mGPDH, iGP-1 (Fig. 4), remained the most potent inhibitor on retesting and also the most selective. The only non-specific effect observed was a weak inhibition of H_2_O_2_ production from site I_Q_ at the low concentration of succinate (see below). In contrast, iGP-2 (Fig. 4) was slightly less potent and had stronger off-target effects on sites I_Q_ and III_Qo_. The end result of our unbiased, multiple parallel chemical screens was a set of novel potent inhibitors of superoxide/H_2_O_2_ production by mGPDH that demonstrated differing selectivity as a result of subtle structural changes. We chose to investigate further the effects of this structural class of compounds on mitochondrial function.

### Analysis of Structure/activity Relationships of Novel mGPDH Inhibitors

Motivated by the observation that subtle changes in structure resulted in changes to both the potency and selectivity of our novel mGPDH inhibitors, we tested an additional 18 compounds structurally related to the top hits in our primary screen to identify structural features that determined the relative potency and selectivity for inhibition of H_2_O_2_ production by mGPDH. These 20 compounds were retested for effects on eight assays of site-specific H_2_O_2_ production and four assays of ΔΨm utilizing different mitochondrial substrates (Fig. 4). To identify structure/activity relationships, compounds were placed into four groups according to common generalized structural differences compared to the original parent compound iGP-1 and evaluated for effects on the 12 assays of mitochondrial function to determine shared effects among group members. Several critical conclusions were drawn from this analysis (Fig. 4). First, as was observed in the original round of retesting described above, changing one of the nitrogen atoms in the benzimidazole to oxygen or sulfur (iGP-2 and iGP-3) had little effect on potency against mGPDH yet decreased selectivity. Specifically, these compounds inhibited H_2_O_2_ production by site I_Q_ to a greater extent and also increased ΔΨm both in the presence and absence of nigericin. These effects on ΔΨm in the presence of nigericin were subsequently found to be caused largely by artifactual quenching of TMRM fluorescence by the compounds (data not shown). However, the much larger increase in ΔΨm observed in the absence of nigericin was found to represent a true change in ΔΨm, most likely a collapse in the ΔpH component of the protonmotive force. This decrease in ΔpH may explain the greater effect of these structural analogs on H_2_O_2_ production by site I_Q_ since this site is known to be uniquely sensitive to ΔpH [44]. Intriguingly, three of four compounds in which additional groups were attached to the free end of the benzimidazole ring were more potent inhibitors of mGPDH (iGP-4– iGP-7). However, these three also had decreased selectivity in similar ways to those observed with changes to the heteroatom of this ring system. The orientation of the benzimidazole and succinamide groups off the central phenyl ring also influenced potency *versus* mGPDH ROS production. Changing the relative positioning of these groups from *ortho-* to *para-* lowered the potency by more than 5-fold (iGP-8). Importantly, altering the carboxyl end of the succinamic acid group decreased potency and selectivity for mGPDH, if any inhibition remained at all (iGP-9– iGP-16). Similarly, the benzimidazole ring was required for inhibition of mGPDH (iGP-17– iGP-20). Ultimately, while our structure/activity analysis yielded no compound with improvements to both potency and selectivity against mGPDH, it provided insight into the structural elements essential to mGPDH inhibition and useful clues as to which chemical features should likely be targeted in future optimization studies.

### iGPs Inhibit mGPDH Enzymatic Activity

**Figure 5:**
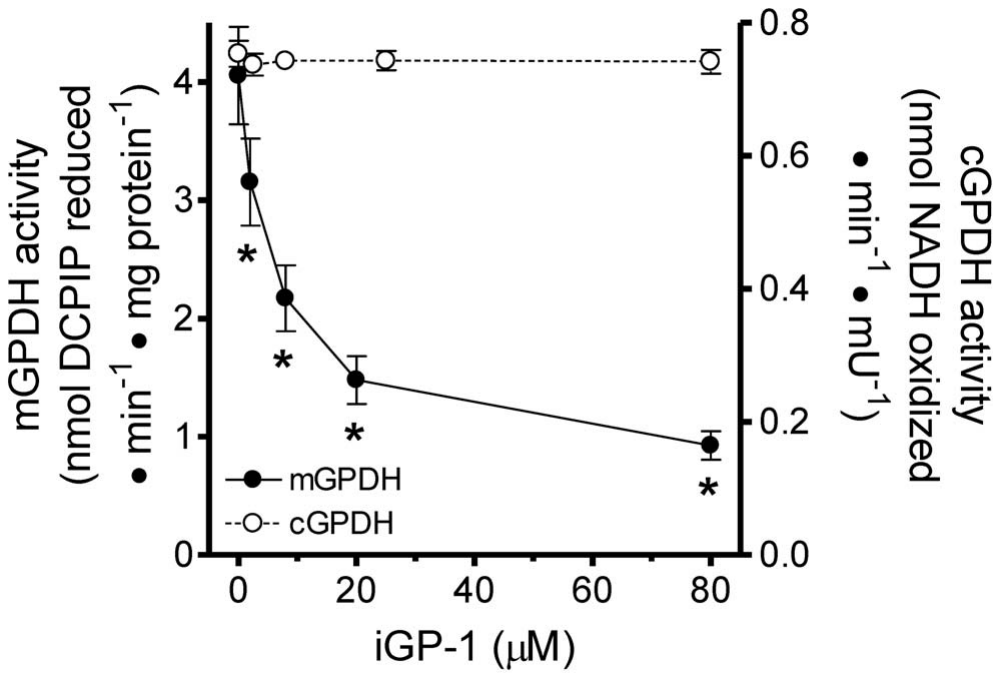
iGP-1 selectively inhibits the mitochondrial isoform of GPDH. Effect of iGP-1 on the enzymatic activities of mGPDH (*black circles*) and cGPDH (*white circles*). iGP-1 significantly inhibited mGPDH activity at all concentrations tested. (*p<0.05 *versus* vehicle control; one-way ANOVA with Newman-Keuls post-test). Data are means ± S.E. (n = 4 for mGPDH, n = 3 for cGPDH).

iGP-1 and iGP-2 did not inhibit ΔΨm driven with glutamate and malate in our initial screen (Fig. 3G). This observation was confirmed during retesting. However, ΔΨm driven by glycerol phosphate was inhibited by several iGPs indicating that these novel compounds inhibited not just H_2_O_2_ production by mGPDH but also its enzymatic activity (Fig. 4). To investigate this distinction, we assayed the effect of iGP-1 on mGPDH enzymatic activity directly. iGP-1 caused significant inhibition of mGPDH enzymatic activity but, remarkably, did not inhibit the soluble form of GPDH (Fig. 5). Next, we further characterized the effects of the most selective, iGP-1, and the most potent, iGP-5, inhibitors of mGPDH on H_2_O_2_ production, ΔΨm, and respiration.

**Figure 6:**
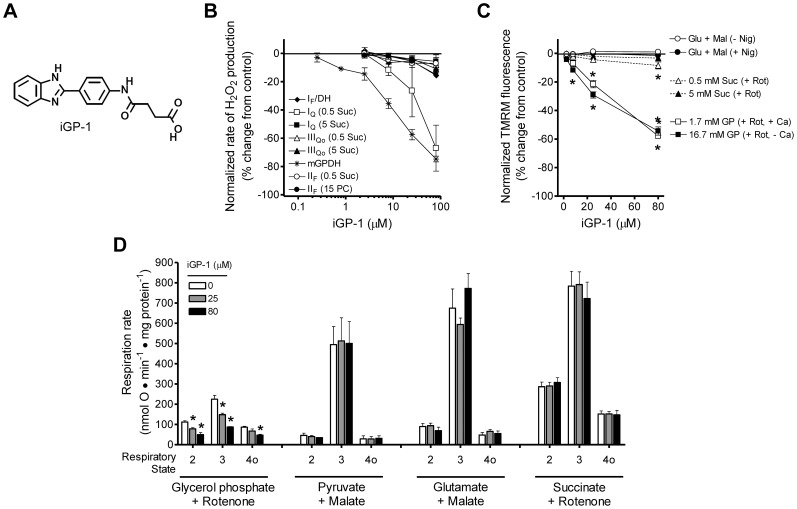
iGP-1 selectively inhibits mitochondrial oxidation of glycerol phosphate. (**A**) Structure of iGP-1. (**B**) Effect of iGP-1 on rates of H_2_O_2_ production from site I_F_/DH (*black diamonds*), site I_Q_ (with 0.5 or 5 mM succinate; *white and black squares*, respectively), site III_Qo_ (with 0.5 or 5 mM succinate; *white and black triangles*, respectively), mGPDH (*black stars*), and site II_F_ (with 0.5 mM succinate or 15 µM palmitoylcarnitine; *white and black circles*, respectively) measured in a fluorimeter. Details of the conditions to induce H_2_O_2_ production from each site are given in Materials and Methods. iGP-1 inhibits H_2_O_2_ production in a site-specific manner from mGPDH. Inhibition of site I_Q_ H_2_O_2_ production driven with 0.5 mM succinate alone (*white squares*) seen at high concentrations of iGP-1 is neither site- nor substrate-selective since site I_Q_ H_2_O_2_ production driven with 5 mM succinate (*black squares*) is not affected and neither site II_F_ nor site III_Qo_ H_2_O_2_ production driven with 0.5 mM succinate (*white circles and triangles*) is altered. Data are normalized means ± S.E. (n = 4 for mGPDH, n = 3 for all other sites). (**C**) Effect of iGP-1 on ΔΨm powered by 5 mM glutamate and 5 mM malate without or with 80 ng ⋅ mL^−1^ nigericin (*white or black circles*), 4 µM rotenone and 0.5 or 5 mM succinate (*white or black triangles*), 1.7 mM glycerol phosphate, 4 µM rotenone, and 250 nM free calcium (*white squares*), or 16.7 mM glycerol phosphate, 4 µM rotenone, and nominal zero free calcium (*black squares*). ΔΨm powered by glycerol phosphate was significantly decreased in the presence of 8, 25, and 80 µM iGP-1. ΔΨm powered by 0.5 mM succinate was significantly decreased by 80 µM iGP-1 whereas no effect was seen on ΔΨm powered by 5 mM succinate or by glutamate and malate. (*p<0.05 *versus* vehicle control; one-way ANOVA with Newman-Keuls post-test). Data are normalized means ± S.E. (n = 3–5). (**D**) Effect of iGP-1 on the rates of mitochondrial respiration driven by 16.7 mM glycerol phosphate with 4 µM rotenone and 250 nM free calcium, 10 mM pyruvate and 0.5 mM malate, 5 mM glutamate and 5 mM malate, or 5 mM succinate and 4 µM rotenone. Respiratory states 2, 3, and 4o were defined by the sequential additions of substrate, 5 mM ADP, and 0.5 µg ⋅ mL^−1^ oligomycin, respectively. iGP-1 significantly decreased glycerol phosphate-dependent respiration at 25 and 80 µM without altering respiration on other substrates. (*p<0.05 *versus* vehicle control; one-way ANOVA with Newman-Keuls post-test). Data are means ± S.E. (n = 3 for pyruvate and malate, n = 5 for all other substrates). No significant effects were observed under any condition with iGP-1 at 2.5 and 8 µM (not shown). *Glu*, glutamate; *Mal*, malate; *Nig*, nigericin; *Suc*, succinate; *PC*, palmitoylcarnitine; *Rot*, rotenone; *GP*, glycerol phosphate; *Ca*, calcium. When not visible, error bars are obscured by the symbol.

The most selective inhibitor, iGP-1 (Fig. 6A), progressively inhibited H_2_O_2_ production by mGPDH as its concentration was increased from 0.25 to 80 µM, with a half-maximal effect at about 8 µM (Fig. 6B). Only above 10 µM did iGP-1 start to inhibit H_2_O_2_ production by site I_Q_, demonstrating its good specificity. This effect on H_2_O_2_ production by mGPDH (Fig. 6B) was mirrored over the same concentration range by significant and specific lowering of ΔΨm driven by glycerol phosphate (Fig. 6C), and significant and specific inhibition of respiratory rates in mitochondria supplied with glycerol phosphate (Fig. 6D), suggesting that iGP-1 inhibited enzymatic activity of mGPDH. iGP-1 decreased H_2_O_2_ production by site I_Q_ and ΔΨm driven by low (0.5 mM) succinate but not by high (5 mM) succinate (Fig. 6B, 6C) indicating a subtle off-target effect on succinate oxidation. It had no effect on H_2_O_2_ production, ΔΨm, or respiration driven by oxidation of a variety of substrates including glutamate, malate, pyruvate, and palmitoylcarnitine.

**Figure 7:**
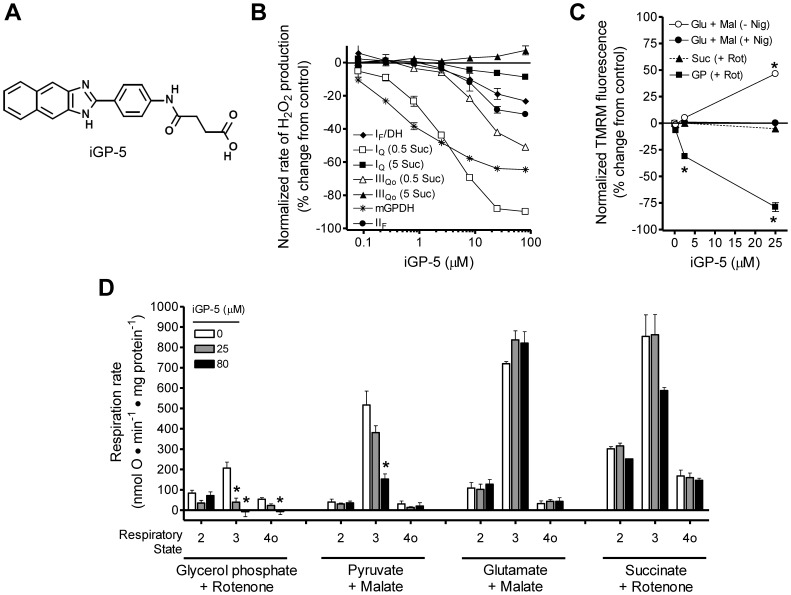
iGP-5 inhibits mGPDH activity more potently than iGP-1 but is less selective. (**A**) Structure of iGP-5. (**B**) Effect of iGP-5 on rates of H_2_O_2_ production from site I_F_/DH (*black diamonds*), site I_Q_ (with 0.5 or 5 mM succinate; *white and black squares*, respectively), site III_Qo_ (with 0.5 or 5 mM succinate; *white and black triangles*, respectively), mGPDH (*black stars*), and site II_F_ (with 15 µM palmitoylcarnitine; *black circles*) measured in microplate format. Details of the conditions to induce H_2_O_2_ production from each site are given in Materials and Methods. iGP-5 potently inhibits H_2_O_2_ production from mGPDH but also causes progressive changes at several other sites of production. Data are normalized means ± ranges (n = 2 technical replicates). (**C**) Effect of iGP-5 on ΔΨm powered by 5 mM glutamate and 5 mM malate without or with 80 ng ⋅ mL^−1^ nigericin (*white or black circles*), 5 mM succinate and 4 µM rotenone (*black triangles*), or 16.7 mM glycerol phosphate and 4 µM rotenone (*black squares*). ΔΨm powered by glycerol phosphate was significantly decreased by 2.5 and 25 µM iGP-5. ΔΨm powered by glutamate and malate was significantly increased by 25 µM iGP-5 but only in the absence of nigericin (*white circles*) suggesting an effect of iGP-5 on the ΔpH component on the proton motive force. (*p<0.05 *versus* vehicle control; one-way ANOVA with Newman-Keuls post-test). Data are normalized means ± S.E. (n = 3). (**D**) Effect of iGP-5 on the rates of mitochondrial respiration driven by 16.7 mM glycerol phosphate with 4 µM rotenone and 250 nM free calcium, 10 mM pyruvate and 0.5 mM malate, 5 mM glutamate and 5 mM malate, or 5 mM succinate and 4 µM rotenone. Respiratory states 2, 3, and 4o were defined by the sequential additions of substrate, 5 mM ADP, and 0.5 µg ⋅ mL^−1^ oligomycin, respectively. iGP-5 significantly decreased glycerol phosphate-dependent state 3 respiration at 25 and 80 µM but also significantly reduced state 3 respiration with pyruvate and malate at 80 µM. (*p<0.05 *versus* vehicle control; one-way ANOVA with Newman-Keuls post-test). Data are means ± S.E. (n = 3). No significant effects were observed under any condition with iGP-5 at 2.5 and 8 µM (not shown). *Glu*, glutamate; *Mal*, malate; *Nig*, nigericin; *Suc*, succinate; *Rot*, rotenone; *GP*, glycerol phosphate. When not visible, error bars are obscured by the symbol.

Similarly, iGP-5 (Fig. 7A) significantly inhibited H_2_O_2_ production by mGPDH as its concentration was increased from 0.08 to 80 µM, with a half-maximal effect at about 1 µM (Fig. 7B). It also lowered ΔΨm (Fig. 7C) and inhibited respiration (Fig. 7D) driven by glycerol phosphate. iGP-5 lowered ΔΨm driven by glycerol phosphate more potently than iGP-1 (Fig. 6C *versus* Fig. 7C). However, this structural analog also displayed less selectivity in assays of H_2_O_2_ production (Fig. 7B) and also significantly increased ΔΨm in the absence but not the presence of nigericin, suggesting a negative effect on ΔpH (Fig. 7C). Further, iGP-5 inhibited respiration driven by pyruvate and perhaps succinate (Fig. 7D).

### iGP-1 is Cell-permeant

**Figure 8:**
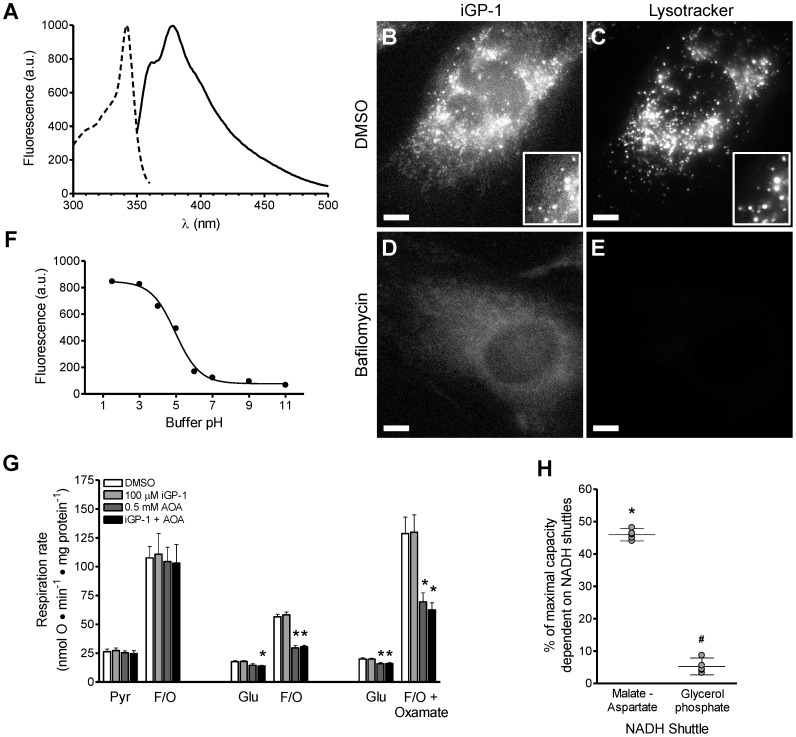
iGP-1 is cell-permeant and inhibits mGPDH in intact presynaptic terminals. (**A**) Excitation and emission spectra of 0.4 mM iGP-1 with peaks at 342 nm and 378 nm, respectively. (**B**) Fluorescence of 100 µM iGP-1 in a live STHdhQ7 cell labeled with LysoTracker Red. (**C**) Fluorescent labeling of acidic vesicles by LysoTracker Red in the same cell shown in (B). Insets in (B and C) highlight colocalization between intense iGP-1 fluorescence and LysoTracker Red labeled vesicles. (**D**) Fluorescence of 100 µM iGP-1 in a live STHdhQ7 cell pretreated with 250 nM bafilomycin A1. (**E**) LysoTracker Red fluorescence in the same cell pretreated with bafilomycin A1 as in (D). Scale bar in (B – E) = 10 µm. (**F**) iGP-1 fluorescence as a function of buffer pH (ex. λ = 342 nm, em. λ = 430 nm). (**G**) Effect of vehicle control (DMSO, *white bars*), 100 µM iGP-1 (*light gray bars*), 0.5 mM aminooxyacetate (*dark gray bars*), or their combination (*black bars*) on synaptosomal respiration. Basal respiration was measured in the presence of either 10 mM pyruvate or 15 mM glucose followed by the addition of 5 µM FCCP and 4 µg ⋅ mL^−1^ oligomycin to induce uncoupled respiration without or with (rightmost condition) 10 mM oxamate. Oxamate was included to minimize regeneration of cytosolic NAD^+^ by lactate dehydrogenase. Respiration on pyruvate alone was not altered by inhibition of either or both of the NADH shuttle systems. Both basal and uncoupled respiration on glucose was unaffected by iGP-1 alone but significantly decreased by aminooxyacetate alone. Oxamate increased the maximal rate of respiration achieved by glucose alone. In the presence of oxamate, the combination of iGP-1 combined with aminooxyacetate consistently decreased the maximal uncoupled rate compared to aminooxyacetate alone. (*p<0.05 *versus* vehicle control and iGP-1 alone; one-way ANOVA with Newman-Keuls post-test). Data are means ± S.E. (n = 3 for pyruvate, n = 4 for glucose, n = 5 for glucose ± oxamate). (**H**) Assignment of the % of maximal respiration that is dependent on the NADH shuttles in the presence of glucose and oxamate. Under this condition of high glycolytic demand, there is a significant dependence upon both the malate-aspartate shuttle and the glycerol phosphate shuttle. (*p<0.05 *versus* no change from vehicle control; ^#^p<0.05 *versus* no change from aminooxyacetate). Data are means ±95% C.I. (n = 5). *AOA*, aminooxyacetate; *Pyr*, pyruvate; *F/O*, FCCP with oligomycin; *Glu*, glucose.

We next determined if iGP-1 was cell-permeant and, therefore, potentially useful for inhibiting mGPDH *in situ*. Several iGPs exhibited fluorescence with excitation maxima between 320–360 nm and emission maxima between 360–480 nm (Fig. 8A and not shown). Cells treated with 100 µM iGP-1 displayed fluorescence above background, with bright puncta in the cytosol, low levels in the nucleus, and intermediate levels distributed diffusely within the cytosol (Fig. 8B). Timelapse imaging of iGP-1 suggested the punctate fluorescence was in rapidly moving structures independent of the mitochondrial network (not shown). Similar distribution of cellular iGP-1 fluorescence was also observed in several other cultured cell lines (e.g. HEK-293 and PC-3). Co-labeling of cells with iGP-1 and LysoTracker Red DND-99, which localizes to acidic vesicles such as endosomes and lysosomes, revealed a high correlation between the most intense iGP-1 fluorescence and acidic vesicles (Fig. 8B and 8C). Treatment of cells with 250 nM bafilomycin A1, an inhibitor of vesicular H^+^-ATPases, collapsed the vesicular pH gradient (demonstrated by loss of LysoTracker Red staining) (Fig. 8E) and also caused loss of the bright, punctate staining of iGP-1 but not the diffuse fluorescence in the cytosol (Fig. 8D). To address whether iGP-1 was accumulated in acidic vesicles or if iGP-1 fluorescence was enhanced at the lower pH of these vesicles, we measured the fluorescence of iGP-1 as a function of pH. We observed an 8-fold increase in iGP-1 fluorescence as the pH was lowered from pH 7 to pH 1.5 (Fig. 8F). Further, addition of the protonophore FCCP to cells caused a rapid, though incomplete, loss of the bright, punctate staining seen with iGP-1 alone (not shown). Together, these data suggest that the intense punctate staining of iGP-1 is likely the result of a pH-dependent enhancement of iGP-1 fluorescence in acidic compartments and not accumulation in vesicles. Importantly, we can conclude that iGP-1 readily crosses cellular membranes and therefore should have access to mGPDH in intact systems.

### Effect of iGP-1 in Cellular Systems

mGPDH is a critical component of the glycerol phosphate shuttle for regenerating cytosolic NAD^+^. However, because of a lack of cell-permeant selective inhibitors of mGPDH and compensation by other NAD^+^ regenerating mechanisms, there is a lack of evidence of functional mGPDH activity in intact systems. We tested the role of mGPDH-dependent metabolism using iGP-1 in synaptosomes because mGPDH is active in neurons [27], [45], [46], there is a significant role for NADH shuttles in synaptic energetics [26], [47], there are no reports on neural function in mGPDH^−/−^ mice, and synaptosomes are a tractable intact system for studying neuronal energetics under tightly controlled cell-, substrate-, and demand-specific conditions.

We verified that the high mGPDH activity reported in brain was present in synaptosomes. In frozen-thawed cortical presynaptic terminals, mGPDH activity was high (207±16 nmol ferricyanide reduced ⋅ min^−1^ ⋅ mg protein^−1^; mean ± SE, n = 3). To demonstrate the likelihood of a functional shuttle system, we measured cGPDH activity in the same preparations. There was a significant rate of DHAP-specific, rotenone-insensitive NADH oxidation that we attribute to cGPDH (22±1 nmol NADH oxidized ⋅ min^−1^ ⋅ mg protein^−1^; mean ± SE, n = 3).

We then applied iGP-1 alone or in combination with aminooxyacetate (to inhibit the malate-aspartate shuttle) (Fig. 1B) in synaptosomes respiring on glucose (Fig. 8G). Maximal demand was created by adding FCCP (plus oligomycin to decrease ATP depletion). In control experiments, pyruvate replaced glucose to bypass the need for NAD^+^ regeneration (Fig. 1). 100 µM iGP-1, 0.5 mM aminooxyacetate, or their combination had no effect on basal or maximal respiration with pyruvate (two left hand sets of columns, Fig. 8G). With glucose as substrate, basal and maximal respiration were inhibited by aminooxyacetate (two middle sets of columns, Fig. 8G), confirming a role for the malate-aspartate shuttle in synaptic bioenergetics [26], [47]. However, iGP-1 had no effect even in combination with aminooxyacetate, suggesting little contribution of the glycerol phosphate shuttle.

In the absence of inhibitors, glucose supported slower respiration than pyruvate. As the result with aminooxyacetate demonstrates, NAD^+^ regeneration has a significant permissive role in synaptosomal metabolism of glucose but not pyruvate. There was also faster extrasynaptosomal acidification with glucose as substrate, suggesting efflux of lactate (with H^+^) to regenerate cytosolic NAD^+^ at lactate dehydrogenase (Fig. 1C). This would divert pyruvate destined for respiration and cause slower respiration with glucose than with pyruvate. To test this hypothesis, we added oxamate (with the FCCP and oligomycin) to inhibit lactate dehydrogenase during oxidation of glucose (Fig. 1C). This should maximally drive respiration while also placing the greatest demand on the NADH shuttles to facilitate glycolysis. Maximal respiration was substantially faster in the presence of oxamate (two right hand sets of columns, Fig. 8G), and aminooxyacetate still inhibited significantly. Importantly, although iGP-1 alone had no effect, it caused consistent additional inhibition in the presence of aminooxyacetate. Thus, there was a significant dependence of uncoupled respiration on both the malate-aspartate shuttle (45.9±0.7%) and the glycerol phosphate shuttle (5.3±0.9%) (means ± SE, n = 5) (Fig. 8H).

### Characterization of the Effects of Novel Inhibitors on the Kinetics of mGPDH

**Figure 9:**
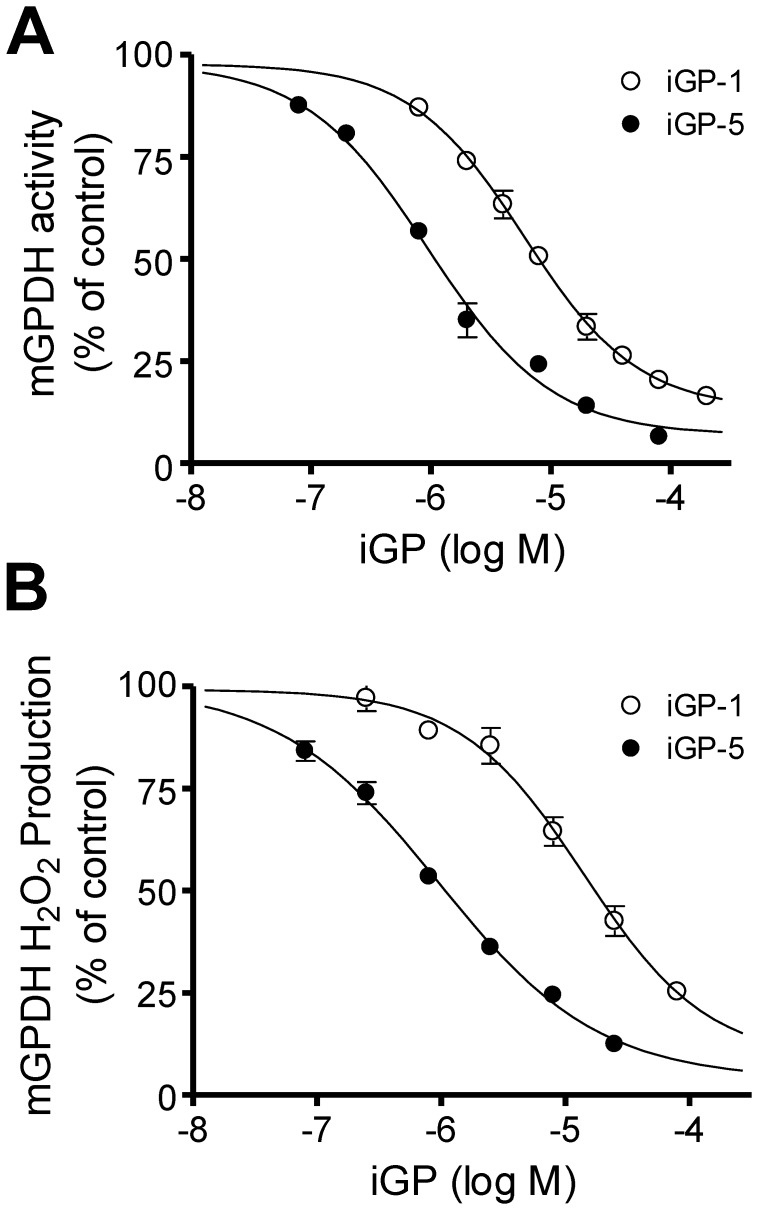
iGP-1 and iGP-5 potently inhibit mGPDH enzymatic activity and H_2_O_2_ production. (**A**) iGP-5 (*black circles*) is a more potent inhibitor of mGPDH enzymatic activity than iGP-1 (*white circles*). IC_50_ concentrations were 1.0 and 6.0 µM for iGP-5 and iGP-1, respectively. Data are means ± S.E. (error bars) (n = 3–5). (**B**) iGP-5 (*black circles*) is a more potent inhibitor of mGPDH H_2_O_2_ production than iGP-1 (*white circles*). IC_50_ concentrations were 1.0 and 14.2 µM for iGP-5 and iGP-1, respectively. Data are means ± S.E. (error bars) (n = 2–4). When not visible, error bars are obscured by the symbol.

**Figure 10:**
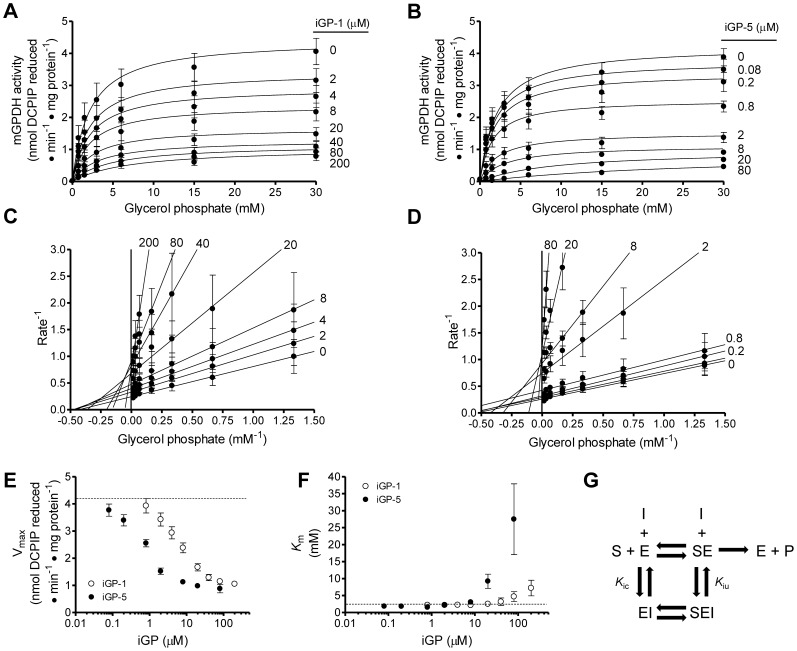
iGP-1 and iGP-5 produce mixed inhibition kinetics. (**A**) Effect of co-varying iGP-1 and glycerol phosphate on mGPDH activity. Curves are hyperbolic best-fits of the Michaelis-Menten equation: mGPDH activity = (Vmax ⋅ [Glycerol phosphate]) ⋅ (*K*_m_+[Glycerol phosphate])^−1^. Data are means ± S.E. (n = 3–4). (**B**) Effect of co-varying iGP-5 and glycerol phosphate on mGPDH activity. Data are means ± S.E. (n = 3–5). (**C**) Double reciprocal plot of the data in (D). Data are means ± S.E. (n = 3–4). (**D**) Double reciprocal plot of the data in (E). Data are means ± S.E. (n = 3–5). (**E**) Effect of iGP-1 (*white circles*) and iGP-5 (*black circles*) on the V_max_ of mGPDH activity. V_max_ values at each concentration of compound were calculated for each experiment using best-fit Michaelis-Menten curves similar to those in (D and E). The dashed line denotes the mean value for the vehicle control. Data are means ± S.E. (error bars) (n = 3–5). (**F**) Effect of iGP-1 (*white circles*) and iGP-5 (*black circles*) on the *K*_m_ of mGPDH. *K*_m_ values at each concentration of compound were calculated for each experiment using best-fit Michaelis-Menten curves similar to those in (D and E). The dashed line denotes the mean value for the vehicle control. Data are means ± S.E. (n = 3–5). (**G**) Scheme for an enzymatic reaction in which enzyme E converts substrate S into product P in the presence of an inhibitor I that displays both competitive and uncompetitive behavior defined by inhibitor dissociation constants *K*_ic_ and *K*_iu_, respectively [43].

The greater potency of iGP-5 *versus* iGP-1 was confirmed against both mGPDH activity (Fig. 9A and Table 1; IC_50_ = 1.0 µM and 6.3 µM, respectively) and H_2_O_2_ production (Fig. 9B and Table 1; IC_50_ = 1.0 µM and 13.6 µM, respectively). Inhibition of mGPDH by both iGP-1 and iGP-5 displayed a selective, one-site binding profile (Table 1; Hill slopes 0.92 and 0.85, respectively). These data support the conclusion that these novel compounds inhibit mGPDH enzymatic activity and confirm that iGP-1 is the most selective of the potent inhibitors we have identified. We next characterized the mechanism of action of this novel class of mGPDH enzymatic inhibitors by measuring the effects on the kinetics of mGPDH. We assayed mGPDH activity at different concentrations of glycerol phosphate and inhibitor (either iGP-1 or iGP-5). These data (Fig. 10A, 10B) showed that the maximum observed rate of DCPIP reduction by mGPDH decreased as the inhibitor concentration increased.

**Table 1:** Inhibition parameters for iGP-1 and iGP-5.

Inhibitor	mGPDH Activity Hill Slope	mGPDH Activity IC_50_ (µM)	mGPDH H_2_O_2_ Production IC_50_ (µM)	*K*_ic_ (µM)	*K*_iu_ (µM)
iGP-1	0.92±0.08	6.3±0.7	13.6±1.3	9.5±1.1	14.6±1.5
iGP-5	0.85±0.07	1.0±0.2	1.0±0.1	0.7±0.0	1.1±0.0

Summary of inhibitor parameters and constants derived from the data presented in Fig. 9 and Fig. 10.

Analysis of double reciprocal plots (Fig. 10C, 10D) showed that each inhibitor lowered the V_max_ and increased the *K*_m_ for glycerol phosphate. Fig. 10E shows that the apparent V_max_ was progressively decreased by each inhibitor with iGP-5 more potent than iGP-1. Fig. 10F shows that the *K*_m_ for glycerol phosphate was progressively increased; again, iGP-5 was more potent than iGP-1. This profile of lowered V_max_ combined with a change in *K*_m_ is indicative of a mixed inhibitor (Fig. 10G) that interacts competitively with respect to the substrate (defined by the competitive inhibitor dissociation constant *K*_ic_) and uncompetitively with the enzyme-substrate complex (defined by the uncompetitive inhibitor dissociation constant *K*_iu_). Values for these dissociation constants were in the 10 µM range for iGP-1 and the 1 µM range for iGP-5 (Table 1).

## Discussion

There is a longstanding need for potent, selective, cell-permeant inhibitors of mGPDH [3], [24]. mGPDH knockout mice indicated a significant role for mGPDH in the survival of nursing pups and in adult adiposity [11], but more effort was required to identify the subtle roles of mGPDH in glucose-stimulated insulin secretion, obligatory thermogenesis, glycerol and fat metabolism, and, specific to mice, liver ureogenesis [12], [48]–[50]. Such long-term studies involving genetic manipulation, condition-dependent phenotypes, and/or pharmacologic interventions are complicated by compensatory mechanisms that mask the involvement of mGPDH. Several 3-carbon glycolytic intermediates, as well as fatty acids and inorganic ions, are known to inhibit mGPDH [2], [13]–[16]. However, many are membrane impermeant, none are selective, and, as we show for the potent competitive inhibitor glyceraldehyde 3-phosphate, can be non-selective even in isolated mitochondria (Fig. 2). Therefore, our novel class of inhibitors offers the first opportunity to acutely test the role of mGPDH activity in a more diverse range of physiological conditions.

Small-molecule screening for modulators of mitochondrial H_2_O_2_ production proved to be an effective strategy for identifying selective inhibitors of mGPDH. The design of the different assays of mitochondrial H_2_O_2_ production and ΔΨm executed in parallel during primary screening and retesting provided multiple filters through which non-selective hits were readily eliminated. Three of the top seven most selective mGPDH inhibitors shared significant structural similarity and the most potent inhibitor in the initial screen, iGP-1, turned out to be the most selective of all the potent analogs identified during subsequent retesting. The design of our screening and retesting strategy also meant that partial selectivity in certain assays yielded insights into potential mechanisms of off-target effects. Combining these insights into an analysis of structure/activity relationships, we revealed that both the succinamic acid and benzimidazole motifs are essential components for mGPDH inhibition by iGPs. Importantly, this analysis identified the benzimidazole ring system as the best candidate for further manipulations to improve both potency and selectivity. In particular, changing or removing the heteroatoms of the imidazole might improve selectivity whereas added substituents to the ring system may provide a means to improve both qualities. We were not able to explore targeted changes to the chemical space occupied by either the linking phenyl group or the succinamide group that did not involve loss of the terminal carboxylic acid. Therefore, these motifs may also provide additional opportunities for improved activity.

Enzyme kinetics revealed that iGPs share a common mechanism of mixed inhibition with respect to glycerol 3-phosphate and that potency was governed by subtle structural changes. Both iGP-1 and iGP-5 lowered the maximal activity of mGPDH and increased the *K*_m_ for glycerol 3-phosphate. Inhibition was enzyme specific, as iGP-1 had no effect on NAD-linked cGPDH activity. This is not surprising considering the two forms are distinct in all respects except their ability to oxidize glycerol 3-phosphate to DHAP. However, this enzyme specificity suggests that iGPs are likely not acting as inhibitory analogs in the substrate binding pocket as described for non-selective inhibitors of both forms such as glyceraldehyde 3-phosphate (data not shown and [2], [13]–[15]). The observation of increased *K*_m_ as well as the lower values for *K*_ic_ than *K*_iu_ indicates that both iGP-1 and iGP-5 have a greater affinity for free enzyme (i.e. mGPDH without glycerol phosphate bound). Both inhibitors have Hill slopes near unity, suggesting they interact with mGPDH at a single, allosteric binding site. Although the analysis of inhibition kinetics was performed in the presence of activating calcium, our evidence from assays of mGPDH-specific H_2_O_2_ production (data not shown) and ΔΨm driven by glycerol phosphate (Fig. 6C) suggest that iGPs act independently of the calcium-sensing mechanism of mGPDH. We cannot rule out direct interactions between iGPs and either the FAD binding domain in the soluble portion of mGPDH or the ubiquinone binding pocket embedded in the outer leaflet of the inner mitochondrial membrane [2]. The interaction with the ubiquinone pocket might be tested by studies similar to those presented here but with differing ubiquinol as the electron donor to the enzyme. Future co-crystallization structural studies and enzymatic assays of iGPs with the bacterial or mammalian FAD-linked GPDH may provide the best opportunities to identify the exact mode of interaction and mechanism of action of these novel inhibitors.

As initial confirmed hits in a small-molecule screen, our most promising iGPs demonstrate excellent potency (IC_50_ values between 1–15 µM) and good selectivity. Apart from a subtle effect on succinate oxidation at high concentrations, iGP-1 does not alter mitochondrial oxidation of numerous substrates including a second dicarboxylate, malate. Therefore, it is unlikely that the subtle effect on succinate oxidation is due to inhibition of the dicarboxylate transporter by the succinamide of iGPs. Indeed, analogs iGP-17– iGP-19 retain the succinamide without or with the attached phenyl ring yet do not alter succinate oxidation as assessed by H_2_O_2_ production by succinate alone (Fig. 4). This suggests at least partial dependence on the benzimidazole ring system for the subtle effect of iGP-1 on succinate oxidation, perhaps *via* direct interaction with complex II. Reactions of the tricarboxylic acid cycle shared between succinate, malate, and pyruvate oxidation also do not appear to be affected by iGP-1. Further, iGP-1 shows no effects on the maintenance of proton motive force or rates of ATP synthesis with substrates other than glycerol phosphate. In addition, we can infer from the synaptosomal experiments that iGP-1 does not prevent pyruvate uptake into cells or mitochondria and does not directly alter glycolysis. Therefore, our data identify an exemplary inhibitor that is both potent and selective against mGPDH and offers structural targets through which additional improvements to these activities can be achieved.

In conclusion, we have identified a novel class of potent, selective, cell-permeant inhibitors of mGPDH that act *via* mixed inhibition. Further tests of the role of mGPDH and glycerol phosphate shuttle activities under conditions of neuronal activity or in other cell types with differing shuttle capacities will help determine those in which mGPDH activity is essential. Our novel inhibitors of mGPDH provide means to test these possibilities pharmacologically.

## Supporting Information

Table S1 Screening results for hits in the mGPDH ROS assay. Summary data for the 87 compounds that selectively inhibited mGPDH ROS. Each value is the average of duplicate “% change from DMSO” determined for each compound at ∼2.5 µM against 6 screening assays.
(XLSX)
